# The cacao Criollo genome v2.0: an improved version of the genome for genetic and functional genomic studies

**DOI:** 10.1186/s12864-017-4120-9

**Published:** 2017-09-15

**Authors:** X. Argout, G. Martin, G. Droc, O. Fouet, K. Labadie, E. Rivals, J.M. Aury, C. Lanaud

**Affiliations:** 1 CIRAD, UMR AGAP, F-34398 Montpellier, France; 2 Commissariat à l’Energie Atomique (CEA), Institut de Génomique (IG) Genoscope, F-92057 Evry, France; 3 Laboratoire d’Informatique, de Robotique et de Microélectronique de Montpellier (LIRMM), CNRS et Université de Montpellier, 34095, Cedex 5 Montpellier, France; 4 Institut de Biologie Computationnelle (IBC), Université de Montpellier, Montpellier, France

**Keywords:** *Theobroma cacao*, Genome Assembly, Mate Paired sequences, GBS, Criollo B97–61/B2 genome

## Abstract

**Background:**

*Theobroma cacao* L., native to the Amazonian basin of South America, is an economically important fruit tree crop for tropical countries as a source of chocolate. The first draft genome of the species, from a Criollo cultivar, was published in 2011. Although a useful resource, some improvements are possible, including identifying misassemblies, reducing the number of scaffolds and gaps, and anchoring un-anchored sequences to the 10 chromosomes.

**Methods:**

We used a NGS-based approach to significantly improve the assembly of the Belizian Criollo B97-61/B2 genome. We combined four Illumina large insert size mate paired libraries with 52x of Pacific Biosciences long reads to correct misassembled regions and reduced the number of scaffolds. We then used genotyping by sequencing (GBS) methods to increase the proportion of the assembly anchored to chromosomes.

**Results:**

The scaffold number decreased from 4,792 in assembly V1 to 554 in V2 while the scaffold N50 size has increased from 0.47 Mb in V1 to 6.5 Mb in V2. A total of 96.7% of the assembly was anchored to the 10 chromosomes compared to 66.8% in the previous version. Unknown sites (Ns) were reduced from 10.8% to 5.7%. In addition, we updated the functional annotations and performed a new RefSeq structural annotation based on RNAseq evidence.

**Conclusion:**

*Theobroma cacao* Criollo genome version 2 will be a valuable resource for the investigation of complex traits at the genomic level and for future comparative genomics and genetics studies in cacao tree. New functional tools and annotations are available on the Cocoa Genome Hub (http://cocoa-genome-hub.southgreen.fr).

**Electronic supplementary material:**

The online version of this article (10.1186/s12864-017-4120-9) contains supplementary material, which is available to authorized users.

## Background

*Theobroma cacao* L. is a tropical fruit tree endemic to the Amazonian basin. It is a diploid species (2n = 2× = 20), with a relative small genome: *T. cacao* genotypes range from 411 Mb to 494 Mb [1]. In the last decade, the genetic diversity and structure of the species has been deciphered using SSR markers resulting in 10 genetic clusters: Amelonado, Contamana, Criollo, Curaray, Guiana, Iquitos, Marañon, Nacional, Nanay and Purús [2]. Several projects have recently been conducted in *Theobroma cacao* genomics to speed up cocoa breeding and to better understand the molecular mechanisms involved in agronomic traits [1, 3, 4].

In 2011, the first *T. cacao* genome of the B97–61/B2 genotype, a member of the Criollo genetic group was released, providing a major source of candidate genes for *T. cacao* improvement and highlighting a genome structure with only slight rearrangements to that of the putative ancestor of the dicotyledon taxon [1]. This draft genome sequence was performed using a whole genome shotgun (WGS) strategy comprising Roche/454 reads for contig assembly, Sanger BAC end sequences for the scaffolding step and a genetic map of 1259 markers to anchor the scaffolds to chromosomes. The final assembly covered 76% of the estimated size of B97–61/B2 and comprised 4792 scaffolds. In addition, 97.8% of the unigenes assembled from the transcriptome data were recovered in the assembly.

Despite the fact that this standard quality draft genome assembly [5] was used as a useful reference, some improvements are still possible, including identifying misassemblies, and reducing the number of scaffolds, gaps, and sequences not anchored to the 10 chromosomes.

In 2013, another WGS project was released for the *Theobroma cacao* Matina1–6 genotype, covering 77% of the evaluated genome size of this member of the Amelonado genetic group [4].

Because they use short sequences from relatively short insert size libraries, any WGS-based de novo sequence assemblies suffer from redundancy due to common repeats such as transposable elements (TEs) and duplicated sequences [6]. As a result, WGS assembly algorithms collapse identical repeats into single regions thereby reducing genomic complexity. Moreover, these collapsed regions should be linked to many other genomic regions and the assembly process will either stop, resulting in a high number of genome fragments, or produce misassembled regions.

It has been found that 41.5% of the Matina1–6 assembled genome was covered by TEs, while B97–61/B2 comprises 35.4% TEs [4].

Today, new methods derived from next generation sequencing (NGS) data are available to improve genome assemblies. Current NGS platforms offer the possibility to produce long reads and positional information using mate-pair templates of large insert size libraries that are capable of spanning many repetitive or low complexity elements in the assembly process [7, 8]. Combined with an accurate gap closing procedure [9], this enables a significant increase in the size of contiguous genomic sequences, a reduction in the number of scaffolds and no discernable misassemblies [10]. NGS-based genotyping has also enabled the discovery of sequence polymorphisms segregating in mapping populations [11]. Recent reports described the use of genotyping by sequencing (GBS) methods to construct dense linkage maps to anchor assembly contigs or scaffolds to chromosomes [10, 12, 13].

In this work, we used a NGS-based approach and significantly improved the assembly of the Belizian Criollo B97–61/B2 genome. We combined four Illumina large insert size mate paired libraries with 52× Pacific Biosciences long reads to correct misassembled regions, to reduce the number of scaffolds and to upgrade their quality. We also used high SNP marker coverage derived from a GBS assay of a UF676 x ICS95 mapping population of 434 individuals and greatly increased the size of the genome sequences anchored to chromosomes.

## Methods

### Sequence data

For this work, we reused some of the data in the dataset produced in the *Theobroma cacao* B97–61/B2 first draft genome project:

1. The 25,912 contig sequences (Acc. number CACC01000000) generated from Roche/454 and Newbler assembler (Roche, Inc) as the initial dataset;
2. The 88,000 Sanger BAC end reads (available on the Cocoa Genome Hub http://cocoa-genome-hub.southgreen.fr) for the scaffolding step;
3. The 398 million Illumina paired end reads as short reads (SR) for error correction (available on the Cocoa Genome Hub http://cocoa-genome-hub.southgreen.fr). Cleaning of SR consisted in: (i) removal of Nextera adapter sequences left in reads, (ii) quality trimming of read extremities (Q > 20), and (iii) discarding reads shorter than 70 bp. All three improvements were performed using a single execution of CutAdapt [14]. The cleaning resulted in 336 million SR for a total of 32 gigabases.

We also generated two new datasets:

1. We created four large insert size mate paired libraries of *Theobroma cacao* B97–61/B2 genome with insert sizes of 3–5 kb, 5–8 kb, 8–11 kb and 11–15 kb using the Nextera Mate Pair Sample Preparation Kit (Illumina, San Diego, CA). These libraries were sequenced by Illumina HiSeq 2000 to respectively 40×, 35×, 19× and 10× genome coverage. The reads were trimmed using the following criteria: (i) sequences of the Illumina adapters and primers used during construction of the library were removed from the whole reads; (ii) nucleotides with a quality value <20 were removed from both ends; (iii) the longest sequence without adapters and low quality bases was kept and the sequence between the second unknown nucleotide (N) and the end of the read was trimmed; (iv) reads shorter than 30 nucleotides after trimming were discarded; (v) finally, reads and their mates that mapped onto run quality control sequences (PhiX genome) were removed. These trimming steps were performed using fastx_clean (http://www.genoscope.cns.fr/fastxtend) based on the FASTX library (http://hannonlab.cshl.edu/fastx_toolkit/index.html).
2. We produced 78 SMRT Cells Pacific Biosciences sequencing data with C2 chemistry that corresponded to 52× genome coverage of long read (LR) data.

Errors in the raw LR dataset were corrected using a hybrid approach by the cleaned SR with LoRDEC [15]. We use a k-mer length of 23 and a solidity threshold of 3. Finally, uncorrected regions at the extremities of LR were trimmed. This yielded 3 million LR with an average length of 2573 bp, representing 21× genome coverage.

### Contigs V1 trimming

*T. cacao* Criollo contig misassemblies were identified using the methods described in detail by [10]. Briefly, large insert size paired reads (LPR) were aligned to contigs using bowtie2 [16] in -very-sensitive mode and misassembly boundaries were identified based on the absence of overlap of read pairs in the region. Misassembled contigs were then split and contigs smaller than 1000 bp were discarded.

Chloroplast and mitochondrion contigs were identified with BLAST [17]. Contigs were aligned to the *T. cacao* Criollo genotype chloroplast genome (acc. no. JQ228379.1). Contigs with at least 98% identity and 80% coverage were discarded for further analysis. In order to identify mitochondrion contigs, the *T. cacao* Criollo contigs were compared to the *Gossypium hirsutum* mitochondrion complete genome (acc. no. JX065074.1).*T. cacao* contigs with an E-value below 1e-40 were discarded.

### Scaffolding

*T. cacao* Criollo contigs were scaffolded using SSPACE [8], with four Illumina large insert size paired reads (LPR) library and the Sanger BAC end sequences generated for the first *T. cacao* Criollo genome assembly [1]. The scaffolding process was performed in five steps, from the shortest inserts library to the longest. Between each step, scaffold misassemblies were identified and resolved based on the absence of overlap of read pairs as described above (see section “Contigs V1 trimming”). To prevent scaffolding errors and because the sequencing depth of the first two LPR libraries was higher than the last two, more stringent parameters were used for the 5 kb and 8 kb LPR (−a 0.5, −k 50) than for the 11 kb and 15 kb (−a 0.5, −k 30). The BAC end sequences were mapped as single end reads using bowtie2 in -very-sensitive mode and read pairs were reconstructed for scaffolding with SSPACE (−a 0.5, −k 5).

### Gap closing

Gaps in scaffolds were closed in two steps. GMcloser [9] was executed in -long_read mode and default option with PacBio error-corrected reads larger than 500 bp. Then, GapCloser [18] was used with the four Illumina LPR libraries with a pair number cutoff for a reliable connection of 5 and a minimum aligned length to contigs for a reliable read location of 35 bp.

### Genetic markers

A total of 434 individuals from the cross between UF676 and ICS95 were sequenced by Diversity Arrays Technology using Illumina HiSeq2000 after DNA restriction with enzymes *Pst*I and *Mse*I. Sequencing fragments were analyzed using Tassel 5 GBS v2.2.24 pipeline [19], and parameter (−mnQS 20). Reads were aligned to scaffolds using Bowtie2 (end-to-end algorithm) and in -very-sensitive mode. Reads that aligned at different locations of the genome were discarded. SNPs were called and variant call data were filtered out with VCFtools [20]. First, indels and non-biallelic sites were excluded. Then, genotyped data with less than 10 reads were recorded as missing data and SNPs with more than 50% of missing data were excluded. Finally, SNPs with a minor allele frequency > 0.01 and *P*-value >1e-6 (**Χ** ^2^ test) were selected for further analysis.

### Scaffold anchoring

The method described by [10] was used to assemble scaffolds into chromosomes. The location of a marker on a scaffold was computed using bowtie2 in -very-sensitive mode. Then pairwise linkage recombination frequencies were calculated between markers with JoinMap4.1 [21] and the resulting data were used to compute order and orientation with an UPGMA like approach.

### Gene annotation

We first used Blastn to transfer the structural annotations from the previously annotated reference genome to the new assembly. For each gene, each exon, extended to 20 bp on both sides, was aligned to the new assembly. We defined drastic criteria (no mismatch and full length alignment) to keep only the complete HSP.

Then, as discrepancies are possible, we performed some quality checks by comparing protein-coding sequences before and after the transfer. For the remaining non-transferred genes, we used Exonerate (cdna2genome model), with the same selection criteria.

Furthermore, a new de novo RefSeq structural annotation, was performed by the NCBI Eukaryotic Genome Annotation Pipeline with the methodology described here https://www.ncbi.nlm.nih.gov/genome/annotation_euk/process/.

Functional annotation was performed with Blastp for each predicted coding sequence against the UniProtKB/Swiss-Prot and UniProtKB/TrEMBL databases [22]. Based on three parameters: (i) Qcov (Query coverage = length high-scoring segment pair (HSP)/length query), (ii) Scov (Subject coverage = length HSP/length subject) and (iii) identity, we kept only the best results to assign a putative function to a polypeptide. InterProScan [23] was used to compare sequences with the InterPro database [24] to obtain additional protein signature information. KEGG pathways [25] have been reconstructed with the BlastKOALA annotation server [26]. The KEEG Orthology assignments were done against the KEGG plant gene database at the genus level.

Additionally, we provided a consensus annotation that combined the NCBI Refseq annotation and the transferred genes from the previously annotated reference genome. Bedtools and custom Perl scripts (with Graph modules) were used to find overlaps between both structural annotations. We kept only the best predictions between both datasets based on RNAseq and functional evidences.

## Results

### Assembly

Since the original *Theobroma cacao* Criollo draft genome was released in 2011 [1], reductions in the cost of sequencing, improved technologies and the publication of the complete *Theobroma cacao* chloroplastic genome [27] as well as release of the complete mitochondrial genome of the closely related species *Gossypium hirsutum* [28] made it possible to update the quality of the *Theobroma cacao* Criollo genome assembly. To reach this goal, we re-scaffolded the original 25,912 contigs of the first version of the *Theobroma cacao* Criollo assembly as described in material and methods.

**Fig. 1:**
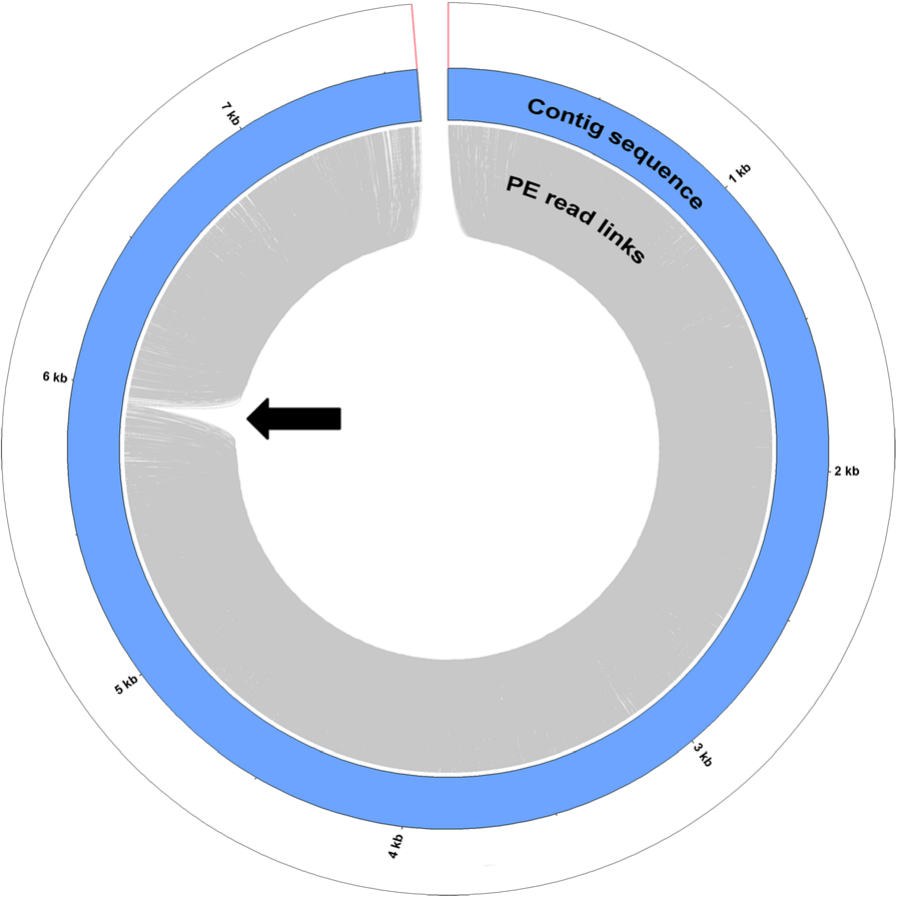
CIRCOS graphical representation of paired reads mapping on a misassembled contig. The blue circle represents the contig sequence. In the inner circle, grey lines represent concordant links (orientation and insert size) between read pairs. The black arrow points to the misassembled region

We first checked the consistency of the original contig dataset by searching for the absence of overlap of read pairs in a region that may result in initial contig assembly errors with the Illumina large insert size libraries (Fig. 1). We discovered that 53 contigs presented a non-overlapped read pair region. These misassembled regions were used to split the corresponding contigs and the new contig dataset created was analyzed to detect organelle sequences.

A total of 37 contigs were detected as chloroplastic and 21 were similar to mitochondrion. These contigs were discarded for the scaffolding step as well as short contigs (<1000 bp). The final clean contigs dataset comprised 25,527 sequences.

### Scaffolding

The 25,527 contigs were scaffolded with SSPACE in a five step process described in the material and methods section. From the shortest insert size library (3–5 kb) to the largest (BAC ends), the number of scaffolded sequences dropped to 554 while the assembly length increased to 325.2 Mb (Table 1). After gap closing, the final assembly V2 comprised 554 scaffolds (versus 4792 in assembly V1) and a total genome length of 324.7 Mb, which represents 75.5% of the estimate size of the B97–61/B2 accession. Fifty percent of the assembly is in 17 scaffolds and the N50 is 6.5 Mb. Gaps in the scaffolds represent only 5.7% of the total assembly.

**Table 1:** Changes in statistics during scaffold assembly

	Library used for assembly	Sequence number	Assembly length (Mb)	N50 (kb)	Unknown (Ns) (Mb)
Contigs	–	25,527	290,5	19,8	–
Scaffolds	3-5 kb	4383	303,9	189,1	13,4
Scaffolds	5-8 kb	1906	312,3	439,4	21,8
Scaffolds	8-11 kb	1271	315,9	709,4	25,4
Scaffolds	11-15 kb	980	318,2	906,5	27,7
Scaffolds	BAC ends	554	325,2	5324,1	34,6
Scaffolds	GapClosure	554	324,7	6465,7	18,5

We then used molecular markers to anchor the scaffolds on the 10 *Theobroma cacao* chromosomes. We used genotyping by sequencing methods to genotype 434 individuals from the UF676xICS95 cross.

A total of 2 × 10^9^ single end Illumina reads were generated and each individual of the progenies produced a mean of 129.5 Mb of high quality sequence. Reads were then aligned to the 554 scaffolds dataset with Bowtie2 and SNP markers were called with the Tassel 5 GBS pipeline. After non-diallelic and indel markers were filtered out, the 434 individuals were genotyped with 39,408 SNP markers. Genotype data with less than 10 reads per data point were recorded as “missing data”. From this raw dataset, we selected a subset of 4857 SNP markers with a percentage of missing data <50% and with a segregation distortion ratio (*P* > 1e-6). The molecular markers were then grouped using JoinMap4.1 software and a linkage group was assigned to each marker. The number of markers per linkage group varied from 694 for linkage group 1 to 298 for linkage group 10 (Table 2).

**Table 2:** Distribution of SNP markers and Scaffolds among *T. cacao* version 2 chromosome assembly

Chromosome	N° of SNP markers	N° of scaffolds	Length (Mb)
Chr1	694	13	37.3
Chr2	581	11	41.2
Chr3	573	15	36.4
Chr4	530	7	31.9
Chr5	597	19	39.4
Chr6	369	13	26.3
Chr7	293	20	21.6
Chr8	321	15	19.6
Chr9	601	13	38.6
Chr10	298	8	21.8
Total	4857	134	314.2

The linkage group information assigned to each SNP marker was first used to verify the consistency of the scaffolds. None of the scaffolds contained molecular markers from a different linkage group, indicating that the scaffolding step was done accurately.

### Chromosome reconstruction and annotation

**Fig. 2:**
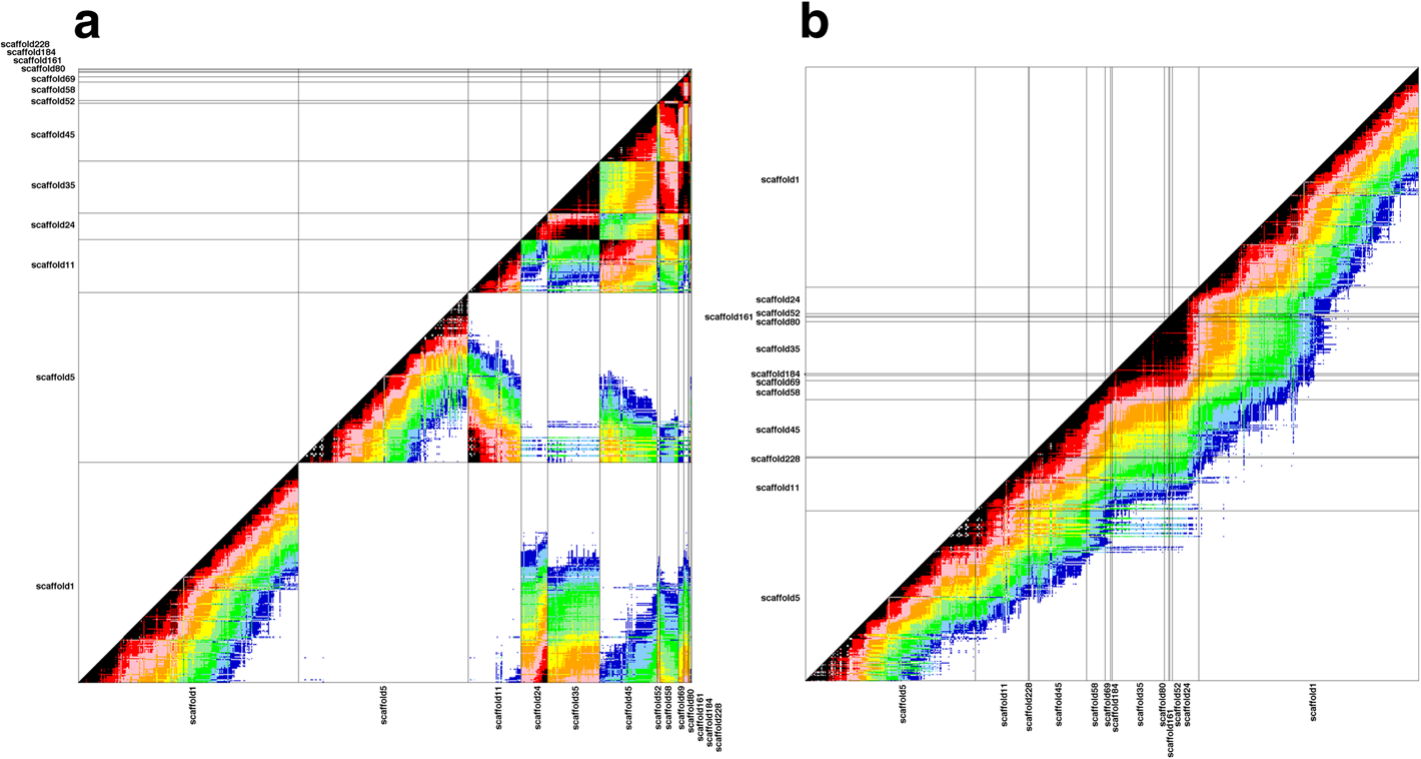
Chromosome reconstruction. Linkage dot plots between markers along non-ordered scaffolds (**a**) and ordered scaffolds (**b**) on chromosome 1. Each dot represents the recombination frequency between two markers. The intensity of the linkage is color coded. Warm colors indicate strong linkage and cold colors indicate weak linkage. Grey bars in the dot plots divide markers belonging to a same scaffold

The pairwise recombination frequencies between each SNP markers were exported from JoinMap4.1 software and used to anchor and orientate the scaffolds to the chromosomes using the method described by [10]. Figure 2 illustrates the process of chromosome reconstruction for chromosome 1. First (Fig. 2a), blocks of already ordered markers were created based on their position on the scaffolds. Then, the recombination frequencies were used to calculate the mean divergence between scaffolds. Finally, the scaffolds were grouped using an UPGMA-like approach and oriented and positioned on a chromosome with a round of optimization (Fig. 2b).

**Fig. 3:**
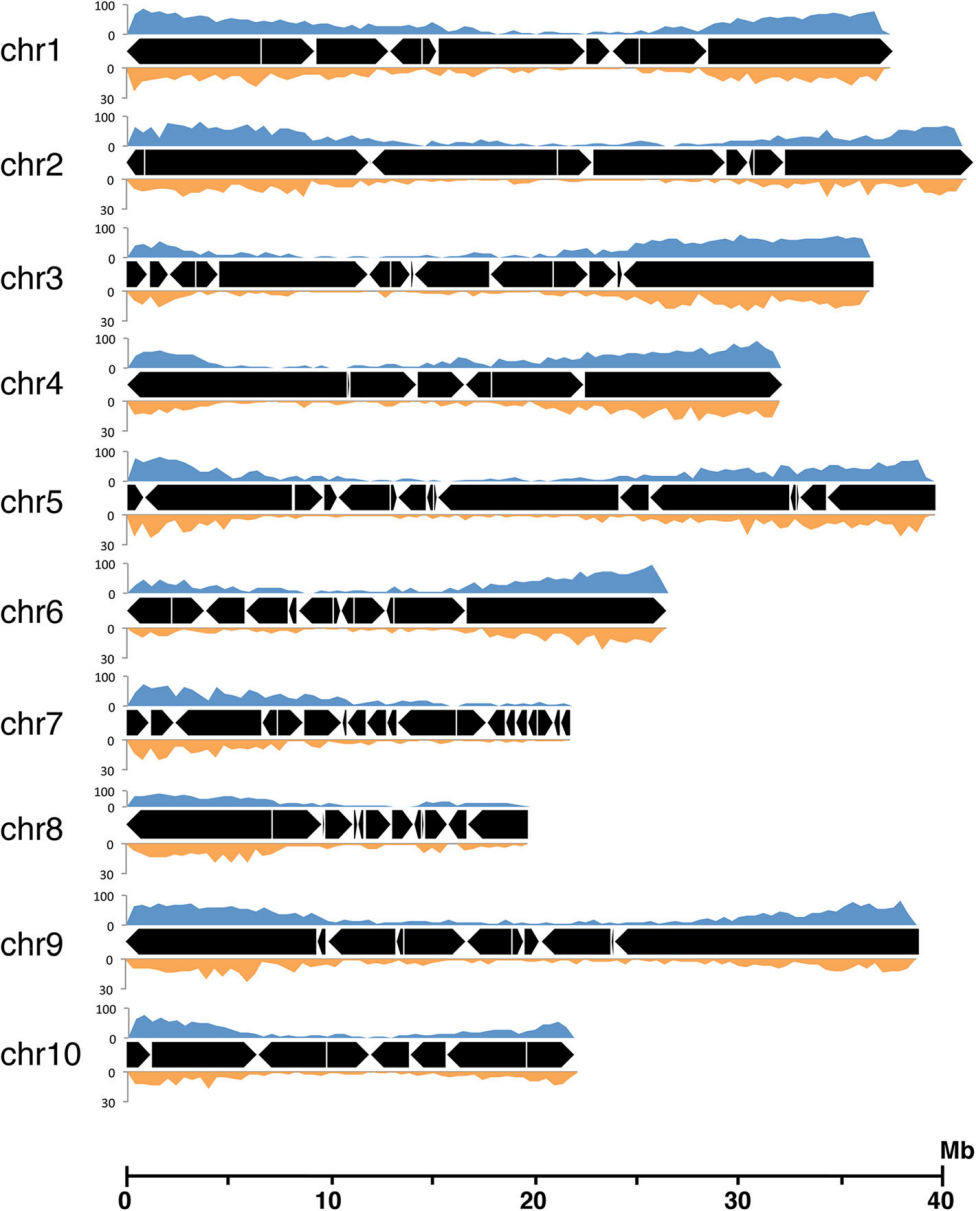
Scaffolds anchored to the 10 *Theobroma cacao* chromosomes. Black boxes represent scaffolds with orientation. Gene and SNP marker densities are in blue and orange, respectively, and were computed with a window size of 400 kb

The process was repeated for the 10 *Theobroma cacao* chromosomes giving a total of 134 scaffolds anchored and oriented to the chromosomes (Fig. 3, Table 2). The number of scaffolds per chromosome ranged from 7 for chromosome 4 to 20 for chromosome 7. The total length of the anchored genome sequence was 314.2 Mb, representing 96.7% of the nuclear genome assembly.

**Fig. 4:**
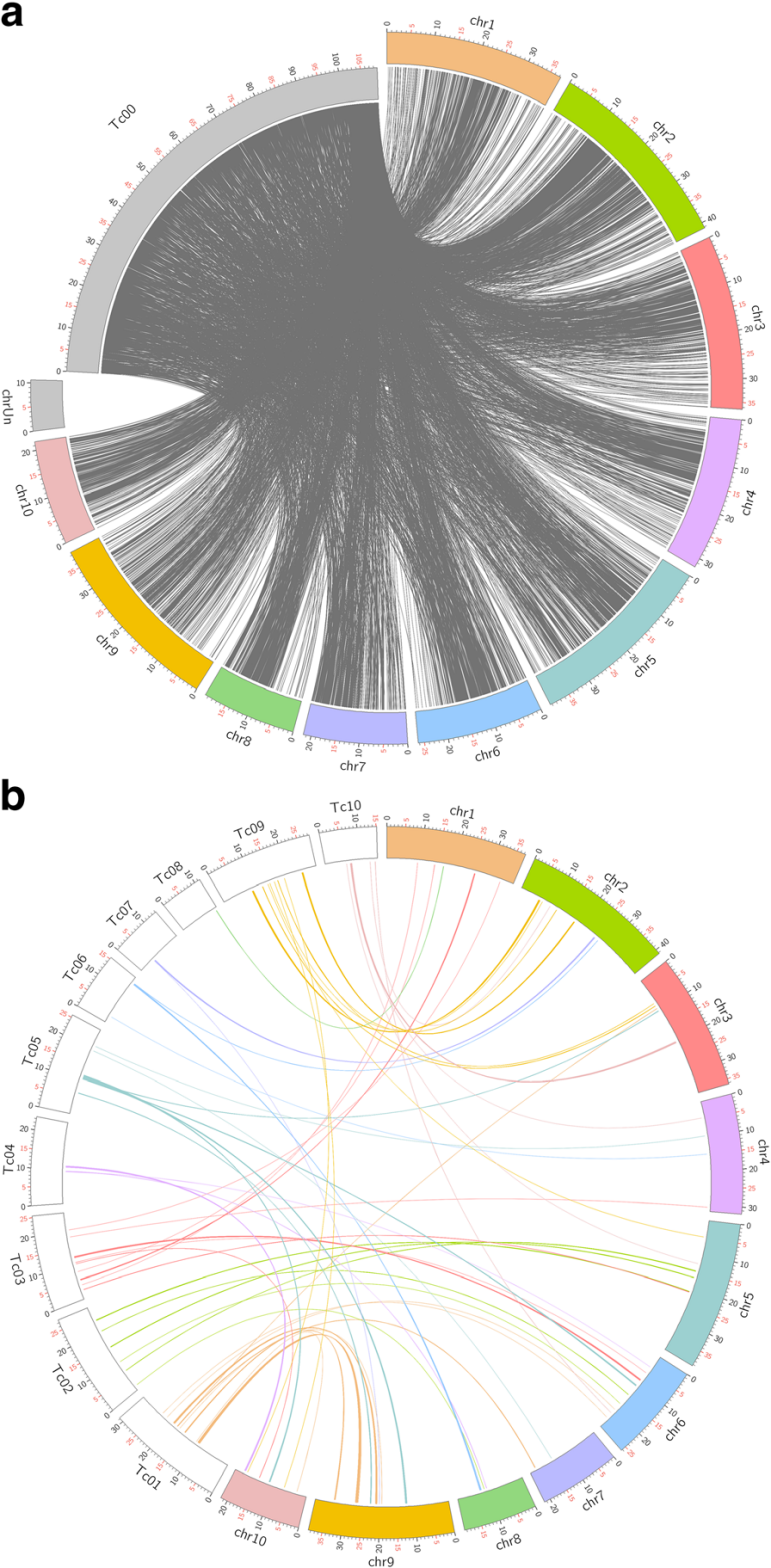
Comparison of *Theobroma cacao* Criollo assembly version 1 and version 2. **a** Graphical representation of insertions and reduction of the unknown chromosome version 1 (Tc00) in chromosomes version 2 (chr1–10). **b** Graphical representation of regions previously anchored to a different chromosome in the first version of the assemblies. “Tc” chromosomes refer to assembly version 1 and “chr” chromosomes to assembly version 2

Compared with the previous version of the Criollo genome assembly, we were able to anchor 95.8 Mb additional DNA sequence to the 10 chromosomes, leading to a significant reduction of the unknown chromosome (Tc00 in the genome v1) (Fig. 4a). We also identified and corrected 45 misassembly points distributed on the 10 chromosomes of the first version of the assembly (Fig. 4b). After gap closing, the proportion of unknown sites (Ns) was reduced from 10.8% in the first version of the assembly to 5.7% in this new assembly (Table 3).

**Table 3:** Statistics for *T. cacao* chromosome assemblies

	B97–61/B2 Version 1 [1]	B97–61/B2 Version 2	Mat 1–6 [4]
Number of scaffolds	4792	554	814
Cumulated size(Mb)	326.9	324.7	346
N50(Mb)	0.47	6.5	4.3
Anchored on chromosomes (Mb)	218.4 (66.8%)	314.2 (96.7%)	330 (95.5%)
Unknown sites (Mb)	35.4 (10.8%)	18.5 (5.7%)	15.2 (4.4%)

### Annotation

The structural annotations of the protein-coding genes computed during the first draft genome project were transferred to the new assembly version. Out of the 28,798 predicted genes, 28,391 (98.6%) were relocated to the assembly version 2. A total of 92% of genes previously located on non-anchored scaffolds (version 1) were transferred to a known chromosome in version 2. In addition, 345 genes from the assembly version 1 were relocated to a different chromosome in assembly version 2. Another structural annotation, supporting evidence from RNA-Seq experiments, was carried out by the NCBI Refseq annotation system. The RefSeq annotation comprised 21,437 protein coding genes, 2229 non-coding genes and 1165 pseudogenes.

We observed in several manually curated genes, that the structural NCBI RefSeq genome annotation was generally of lower quality than the first published annotation in particular because the NCBI RefSeq tended to merge adjacent gene models. Otherwise the NCBI RefSeq annotation integrated RNAseq data and predicted alternative transcripts and could be a useful resource in gap closed regions where no genes were predicted by the first annotation system. Therefore, we made a consensus annotation to select the best structural predictions between both datasets as described in material and methods. The final consensus annotation comprised 29,071 protein coding gene models.

The functional annotation was carried out for the three structural annotations. The full annotations as well as a genome browser are available through the Cocoa Genome Hub (http://cocoa-genome-hub.southgreen.fr).

## Discussion

The rapid advances in NGS-based methods since the first draft genome sequence of *Theobroma cacao* Criollo B97–61/B2 was published in 2011 [1] offered an opportunity to update the quality of the genome assembly.

During the first steps of the scaffolding process, the four mate-paired libraries reduced the number of scaffolds to 980, which is 80% less than in the first published version of the genome (4792) and doubled the N50 size value (932 kb vs. 470 kb). The addition of BAC end sequences enabled us to reduce the number of scaffolds by almost 90% (from 4792 to 554) and increase the N50 size value 14 fold (from 0.47 to 6.5 Mb). Our result demonstrates the usefulness of large size mate pair templates to correct misassemblies and to reduce the number of scaffolds and consequently increase the size of contiguous sequences.

In the final assembly, we closed almost half the gaps (34.6 Mb vs. 18.5 Mb) with a combination of long read sequences and large insert size mate paired libraries. After error correction, gaps closed by Pacific Bioscience reads represent 4.4 Mb of sequence. The LR data would have had a bigger impact if they were generated with the P6-C4 chemistry, which yields longer average read lengths than C2 chemistry (average length of 2573 nucleotides of the corrected LR dataset used in this study). In addition, the absence of non-overlapped read pair regions in the gap filled scaffolds confirmed the efficiency of the gap closure step.

The cumulative size of the new assembly (324.7 Mb) is reduced by 2.2 Mb in comparison with the first genome assembly. This reduction is mainly due to the better efficiency of large size mate pair templates to evaluate gap size during scaffolding process.

The power of genotyping by sequencing methods to produce a high number of SNP molecular markers was used to increase the proportion of the assembly anchored to chromosomes to 96.7% compared to 66.8% in version 1. Moreover, 99% of the genes are now anchored to chromosomes compared to 82% in the first assembly. Many genes previously described to be involved in *Theobroma cacao* agronomical traits [1] and not assigned to chromosomes in version 1 of the genome are now located on chromosomes in version 2. For instance, in genome version 1, 49 disease resistance-related genes orthologous to *Arabidopsis LRR-RLK* genes were located to non anchored regions. In *T. cacao* genome version 2, only 8 genes remain located in unanchored regions. Similarly, among the 57 terpenoïd-encoding genes involved in biosynthesis pathway of aromas, only 2 genes in version 2 (14 in version 1) are not located to known chromosomes. Therefore, the improvement of the amount of sequences anchored to the chromosomes leads to a better genomic framework that could be used for a better elucidation of QTLs by mapping agronomical traits and identifying candidate genes within a region of interest.

**Fig. 5:**
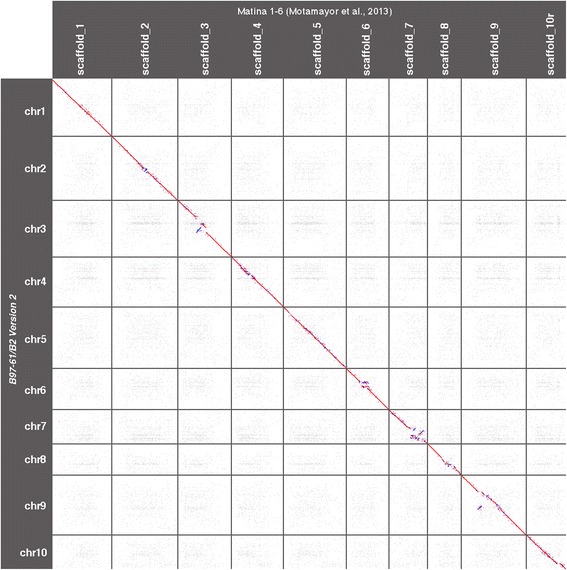
Dot plot comparing Criollo B97–61/B2 version 2 and Amelonado Matina 1–6 genomes computed with Last [29]. Red and blue dots indicate forward and reverse alignments, respectively

Comparison of the sequences of this new version of the B97–61/B2 genome with the Matina1–6 genome revealed good collinearity between the two genome assemblies (Fig. 5). The main observed differences were scaffold inversions located in peri-centromeric regions of chromosomes 2, 6, 7, 8 and 9.

Because both assemblies were anchored to chromosomes using genetic information and because a low recombination rate is observed in centromeric regions (Additional file 1: Figure S1), these differences can be explained by the difficulty involved in ordering and orienting scaffolds in these regions where genotyping errors could be considered by the algorithm as recombination events.

This better chromosome-scale assembly of the Criollo genome could help to identify syntenic blocks shared between *T.cacao* genome and related species. Combined with genetic studies, comparative genomics studies could enable researchers to identify genes, regulatory elements, noncoding RNAs, and conserved sequences of unknown function involved in *T. cacao* agronomic traits.

## Conclusions

Genome assembly is a dynamic and continuous process that can be improved by advances in sequencing technologies and methods. The correction of misassemblies, resolution of gaps, reduction in the number of scaffolds and non-anchored regions and updates of the functional and structural annotations we made to the first published *Theobroma cacao* genome sequence is an important step forward for future comparative genomics and genetic studies in cocoa.

## Additional file

Additional file 1:
**Figure S1.** Recombination rates (cM/Mb) computed with the MareyMap R package [30]. (PNG 1418 kb)

## Abbreviations

BAC: Bacterial artificial chromosome
GBS: Genotyping by sequencing
LPR: Large insert size paired read
LR: Long read
NGS: Next generation sequencing
SNP: Single nucleotide polymorphism
SR: Short read
TE: Transposable element
WGS: Whole genome shotgun
