# Supplementary figures and images for: The cacao Criollo genome v2.0: an improved version of the genome for genetic and functional genomic studies
Source: BMC Genomics. 2017 Sep 15;18:730. doi: 10.1186/s12864-017-4120-9 (PMC5603072; doi:10.1186/s12864-017-4120-9)

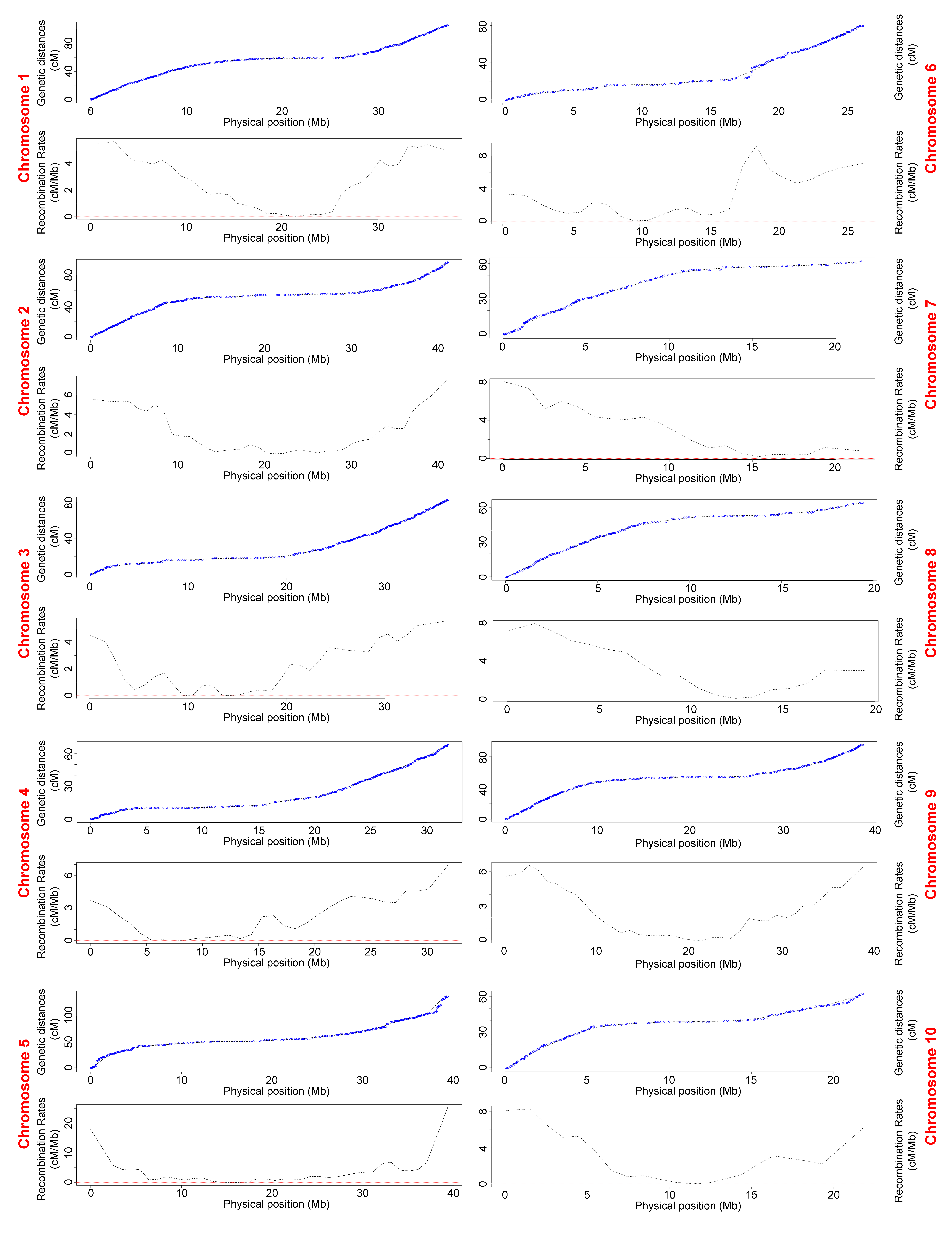

Supplement: Additional file 1: — Figure S1. Recombination rates (cM/Mb) computed with the MareyMap R package [30]. (PNG 1418 kb) [file 12864_2017_4120_MOESM1_ESM.png]
